# Cellular scaling rules for the brain of afrotherians

**DOI:** 10.3389/fnana.2014.00005

**Published:** 2014-02-17

**Authors:** Kleber Neves, Fernanda M. Ferreira, Fernanda Tovar-Moll, Nadine Gravett, Nigel C. Bennett, Consolate Kaswera, Emmanuel Gilissen, Paul R. Manger, Suzana Herculano-Houzel

**Affiliations:** ^1^Instituto de Ciências Biomédicas, Universidade Federal do Rio de JaneiroRio de Janeiro, Brasil; ^2^Instituto Nacional de Neurociência Translacional, CNPq/MCTSão Paulo, Brasil; ^3^D'Or Institute for Research and Education (IDOR)Rio de Janeiro, Brasil; ^4^Bioimaging National Center, Federal University of Rio de JaneiroRio de Janeiro, Brazil; ^5^Department of Zoology and Entomology, University of PretoriaPretoria, South Africa; ^6^Faculté des Sciences, University of KisanganiKisangani, Democratic Republic of Congo; ^7^Department of African Zoology, Royal Museum for Central AfricaTervuren, Belgium; ^8^Laboratory of Histology and Neuropathology, Université Libre de BruxellesBrussels, Belgium; ^9^Department of Anthropology, University of ArkansasFayetteville, AR, USA; ^10^School of Anatomical Sciences, University of the WitwatersrandJohannesburg, South Africa

**Keywords:** evolution, glia-neuron ratio, numbers of neurons, cortical expansion, gyrification

## Abstract

Quantitative analysis of the cellular composition of rodent, primate and eulipotyphlan brains has shown that non-neuronal scaling rules are similar across these mammalian orders that diverged about 95 million years ago, and therefore appear to be conserved in evolution, while neuronal scaling rules appear to be free to vary in evolution in a clade-specific manner. Here we analyze the cellular scaling rules that apply to the brain of afrotherians, believed to be the first clade to radiate from the common eutherian ancestor. We find that afrotherians share non-neuronal scaling rules with rodents, primates and eulipotyphlans, as well as the coordinated scaling of numbers of neurons in the cerebral cortex and cerebellum. Afrotherians share with rodents and eulipotyphlans, but not with primates, the scaling of number of neurons in the cortex and in the cerebellum as a function of the number of neurons in the rest of the brain. Afrotheria also share with rodents and eulipotyphlans the neuronal scaling rules that apply to the cerebral cortex. Afrotherians share with rodents, but not with eulipotyphlans nor primates, the neuronal scaling rules that apply to the cerebellum. Importantly, the scaling of the folding index of the cerebral cortex with the number of neurons in the cerebral cortex is not shared by either afrotherians, rodents, or primates. The sharing of some neuronal scaling rules between afrotherians and rodents, and of some additional features with eulipotyphlans and primates, raise the interesting possibility that these shared characteristics applied to the common eutherian ancestor. In turn, the clade-specific characteristics that relate to the distribution of neurons along the surface of the cerebral cortex and to its degree of gyrification suggest that these characteristics compose an evolutionarily plastic suite of features that may have defined and distinguished mammalian groups in evolution.

## Introduction

Mammalian brain size varies by a factor of approximately 100,000 across species (Tower, 1954; Stolzenburg et al., 1989). Comparative studies have traditionally used volume or surface measurements to investigate this variation (Jerison, 1985; Haug, 1987; Zhang and Sejnowski, 2000), resting upon the assumption that, regarding cellular composition, all mammalian brains are scaled versions of the same model. Should that be the case, surface and volume would work as indirect measures of cellular composition, which relates to the computational capacity of brains (Williams and Herrup, 1988).

In contrast, a recent methodological development - the isotropic fractionator (Herculano-Houzel and Lent, 2005)—allows for the absolute number of neurons and non-neurons in anatomically defined brain regions to be readily quantified. This is a non-stereological method that yields similar results to stereology in a smaller amount of time (Bahney and von Bartheld, 2014). Studies employing the isotropic fractionator have revealed that cellular scaling rules are different both across different structures within the same brain and across mammalian orders, making the use of volume or surface as a proxy for cellular composition misleading (Herculano-Houzel et al., 2006, 2007; Sarko et al., 2009; Gabi et al., 2010).

The picture emerging from the isotropic fractionator studies is one where the mass of a structure varies as markedly different functions of its numbers of neurons across orders and structures (that is, the neuronal scaling rules differ across brain structures and mammalian orders). On the other hand, structure mass varies as a similar, shared function of its number of non-neurons across orders and structures (that is, the non-neuronal scaling rules are shared; reviewed in Herculano-Houzel, 2011a). A limitation of these investigations to date is that the groups of mammals investigated (Rodentia, Primata, and Eulipotyphla) are phylogenetically close. While it is remarkable to find such diversity in the neuronal scaling rules across orders that are closely related, this phylogenetic affinity does not reveal how universal the non-neuronal scaling rules may be across mammals as a whole.

The mammalian superorder Afrotheria is especially interesting to investigate because of its key phylogenetic origin at the base of the placental mammals tree (Murphy et al., 2007). Afrotherians are a heterogeneous clade of mammals that diverged from the previously investigated groups over 100 million years ago (Stanhope et al., 1998; Springer et al., 2004; Tabuce et al., 2007; Poulakakis and Stamatakis, 2010; Kuntner et al., 2011). Its members include animals as diverse as elephants, hyraxes, sea cows and dugongs, aardvarks, tenrecs, elephant shrews and golden moles. They inhabit a wide variety of habitats and present a large range of variation in both brain and body size.

Due to morphological and ecological similarities, some of the small afrotherians were, until recently, classified under the Insectivora umbrella, along with species that were later moved to the order Eulipotyphla (Stanhope et al., 1998). Because of the ecological similarities across small afrotherians and Eulipotyphla, a comparison of these small mammals might give insight into the balance between ecological and phylogenetic constraints in brain evolution.

Here we determine the number of neurons and other cells in the brains of 5 small-bodied species of Afrotheria, using the isotropic fractionator (Herculano-Houzel and Lent, 2005). We investigate then how the cellular scaling rules for these mammals relate to previously found scaling rules, in particular the ecologically similar Eulipotyphla.

## Methods

### Animals

Adult eastern rock elephant shrews (*Elephantulus myurus*, *n* = 2), four-toed elephant shrews (*Petrodromus tetradactylus*, *n* = 2), golden moles (*Amblysomus hottentotus*, *n* = 2), rock hyraxes (*Procavia capensis*, *n* = 2) and western tree hyrax (*Dendrohyrax dorsalis*, *n* = 1) were caught from field populations. Appropriate permissions to trap and euthanize these mammals were obtained from the provincial Departments of Nature Conservation, South Africa, and the University of Kisangani, Democratic Republic of the Congo. All animals were treated and used in accordance with the University of the Witwatersrand Animal Ethics Committee Guidelines (clearance number 2008/36/1) which parallel those of the NIH for the care and use of animals in scientific experiments.

### Dissection

All animals were euthanized (overdose of sodium pentobarbital, 100 mg/kg, i.p.) and perfused through the left ventricle with 0.9% saline, followed by 4% paraformaldehyde in 0.1M phosphate buffer (PB, pH 7.4). Following perfusion, the brains were removed, weighed, and post-fixed in 4% paraformaldehyde in 0.1 M PB overnight, cryoprotected in 30% sucrose in 0.1 M PB at 4°C and stored in an antifreeze solution at −20°C until processing. The brains were divided into two halves along the mid-sagittal fissure and one hemisphere of each brain processed. The cerebellum was dissected by cutting the cerebellar peduncles at the surface of the brainstem. The cerebral cortex in all animals was manually dissected from the striatum and other subcortical structures. The hippocampus was then dissected from each cortical hemisphere, under a stereoscope. The cerebral cortex of the hyrax specimens was then cut into 2 mm coronal sections in order to allow the dissection of gray and white matter, which had their numbers of cells counted separately. The olfactory bulbs, when available, were also dissected and weighed individually. All other brain structures were pooled and processed together as “rest of brain.”

Since only one hemisphere of each brain was available for analysis, values reported here are multiplied by 2 to give estimates for the whole brain that are comparable with our previously published data (Herculano-Houzel et al., 2006, 2007, 2011; Sarko et al., 2009; Gabi et al., 2010). For the sake of consistency with our previous studies, and because the olfactory bulb was not available for all specimens, whole brain values used in the analysis exclude the olfactory bulb.

### MRI and morphometry

Prior to dissection, magnetic resonance imaging (MRI) was performed in the whole hemisphere of each analyzed brain. Images were acquired in a 7-T magnetic resonance scanner (MRI System 7T/210 ASR Horizontal Bore Magnet, Agilent Technologies) located at the Bioimaging National Center, Federal University of Rio de Janeiro, Brazil. The imaging protocol included a FLAIR sequence (TR/TE: 800/15.50 ms; matrix: 192 × 192, slice thickness: 1 mm; 6 averages; field of view: 50 × 80 cm). Care was taken to ensure that the whole brain hemisphere was scanned and to avoid partial volume effect on the first and last slices.

Data processing was performed using *Osirix* (www.osirix-viewer.com) and NeuroLucida software (MBF Bioscience, Vermont, USA). Prior to analysis, all images were visually inspected for artifacts. For each MRI data set, the gray and white matter boundary was manually traced in all slices and the total gray and white matter volumes were then computed.

### Isotropic fractionation

Total numbers of cells, neurons, and non-neuronal (other cells) were estimated as described previously using the isotropic fractionator method (Herculano-Houzel and Lent, 2005). Briefly, this method turns each dissected brain division into an isotropic suspension of isolated nuclei of known, defined volume, kept homogeneous by agitation. The total number of nuclei in suspension—and therefore the total number of cells in the original tissue—is estimated by determining the density of nuclei in small aliquots stained with the fluorescent DNA marker DAPI (4′-6-diamidino-2-phenylindole dihydrochloride, Invitrogen, USA) under the microscope.

For each structure, at least 4 samples of the nuclear suspension are counted independently, in different chambers of the hemocytometer, to determine the number of nuclei/ml of the suspension. The reported values for total number of cells refer to the average nuclei/ml of the samples taken multiplied by the total volume of the suspension. This consistently yields a coefficient of variation of 0.10 or less, and never more than 0.15, across samples from the same structure.

Once the total cell number in a structure is known, the proportion of neurons is determined by immunocytochemical detection of neuronal nuclear antigen (NeuN, Millipore mab377), expressed in the nuclei of most neuronal cell types and not in non-neuronal cells (Mullen et al., 1992; Gittins and Harrison, 2004). Estimates of the proportion of NeuN-positive nuclei are considered reliable since the coefficient of variation among animals of the same species is typically below 0.15. Numbers of other cells are derived by subtraction.

### Data analysis

All statistical analyses and regressions were performed in JMP 9.0 (SAS Institute, North Carolina), using the average values obtained for each species. Spearman rank correlations were calculated for each relationship, and when significant, regressions to power and linear functions were performed to find the best fit for the distribution.

For the comparison with cellular scaling rules reported previously, we used the equations that apply to the average structure size and cellular composition for the species of the groups described earlier: Primata (Gabi et al., 2010), excluding tree shrews; Glires (Herculano-Houzel et al., 2006, 2011); and Eulipotyphla (Sarko et al., 2009), as reviewed in Herculano-Houzel (2011a).

### Results

**Figure 1:**
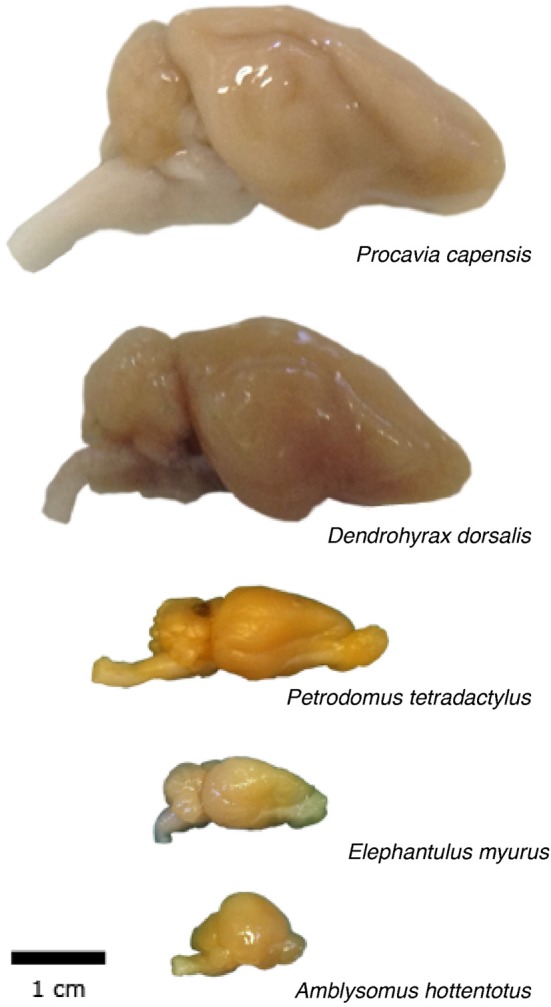
**Overview of the morphology of the afrotherian brains used in the study**.

**Figure 2:**
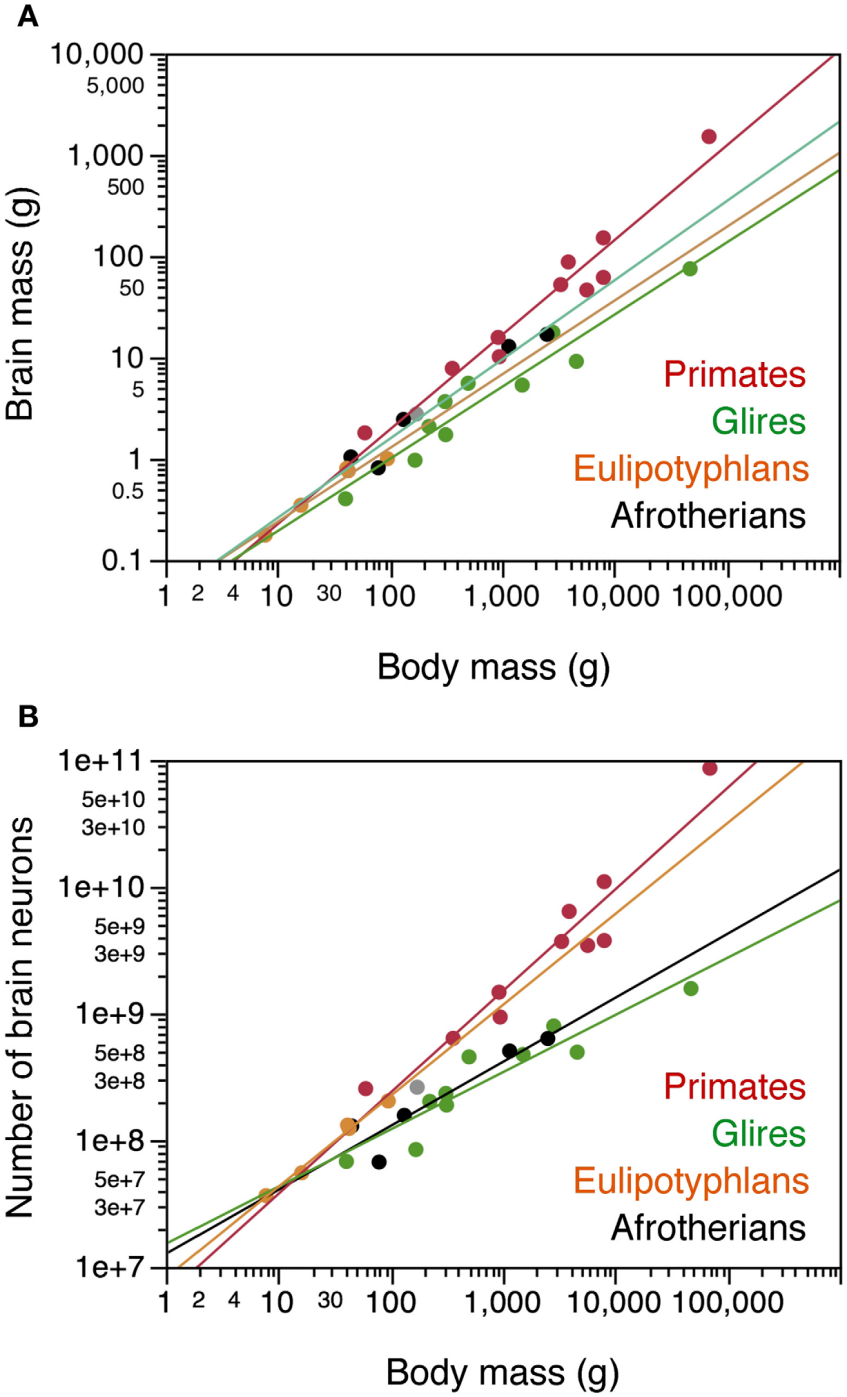
**Afrotherian brains gain mass similarly to Glires and eulipotyphlans, but gain neurons similarly to Glires and not to eulipotyphlans or primates**. Scaling relationships for afrotherian brains (in black) are plotted along with rules found previously for other groups (primates in red, glires in green, eulipotyphlans in orange, scandentia in gray). Graphs relate **(A)** total brain mass (excluding the olfactory bulb) and **(B)** total numbers of neurons in the brain (excluding the olfactory bulb) to body mass. Each point represents the average values for each species. Data from Herculano-Houzel et al. (2006, 2007, 2011); Azevedo et al. (2009); Sarko et al. (2009); Gabi et al. (2010). Power functions for each group are plotted. Exponents for brain mass × body mass are 0.781 ± 0.101 (afrotherians), 0.712 ± 0.071 (Glires), 0.727 ± 0.094 (eulipotyphlans), 0.934 ± 0.068 (primates with Scandentia). Exponents relating number of brain neurons to body mass are 0.505 ± 0.113 (afrotherians), 0.451 ± 0.061 (Glires), 0.717 ± 0.045 (eulipotyphla), and 0.801 ± 0.084 (primates).

Across the 5 species analyzed here (Figure 1), average body mass varies 55.8-fold (from 45 g in the elephant shrew to 2.5 kg in the rock hyrax), while brain mass varies 20.8-fold, and total number of brain neurons varies only 9.4-fold (Table 1). In agreement with the faster increase in body mass (*M*_BD_) than in brain mass (*M*_BR_), we find that brain mass increases as a power function of body mass with an exponent smaller than 1.0 such that *M*_BR_ = 0.0430 × *M*_BD_^0.781±0.101^ (*p* = 0.0045; Figure 2A). This relationship overlaps with those found previously for rodents and eulipotyphlans, which, like afrotherians, have smaller brains than primates of a similar body mass (Figure 2A). The total number of brain neurons (not including the olfactory bulb) also increases as a power function of body mass, with an even smaller exponent of 0.505 ± 0.113 (*p* = 0.0208; Figure 2B). This relationship also overlaps with that found previously for rodents, but not eulipotyphlans, which overlap with primates (Figure 2B).

**Table 1:** **Cellular composition of afrotherian brains**.

	***E. myurus* (*n* = 2)**	***P. tetradactylus* (*n* = 2)**	***A. hottentotus* (*n* = 2)**	***P. capensis* (*n* = 2)**	***D. dorsalis* (*n* = 1)**
*M*_BD_	45.08	132.50	79.00	2517.00	1150.00
*M*_BR_	1.040 ± 0.082	2.440 ± 0.109	0.812 ± 0.044	16.853 ± 1.495	12.800
*M*_CX_	0.355 ± 0.009	0.968 ± 0.076	0.388 ± 0.024	9.184 ± 0.182	6.830
*M*_HP_	0.116 ± 0.030	0.271 ± 0.017	0.051 ± 0.011	1.294 ± 0.464	0.730
*M*_CxT_	0.471 ± 0.021	1.239 ± 0.059	0.439 ± 0.035	10.478 ± 0.646	7.560
*M*_CB_	0.168 ± 0.002	0.304 ± 0.028	0.084 ± 0.004	2.058 ± 0.224	1.920
*M*_RoB_	0.401 ± 0.063	0.894 ± 0.022	0.289 ± 0.013	4.317 ± 0.625	3.330
*M*_OB_	0.050 ± 0.010	0.159 ± 0.009	0.050	0.290	n.a.
*N*_BR_	129.190 ± 4.424	156.831 ± 20.600	65.074 ± 2.124	755.653 ± 72.145	504.564
*N*_CX_	16.423 ± 0.265	27.652 ± 6.497	19.870 ± 2.103	171.209 ± 35.277	90.650
*N*_HP_	9.443 ± 4.284	6.294 ± 0.658	1.646 ± 0.500	26.712 ± 6.216	8.313
*N*_CxT_	25.865 ± 4.020	33.947 ± 5.840	21.516 ± 2.154	197.933 ± 29.082	98.960
*N*_CB_	89.312 ± 2.852	110.653 ± 14.948	34.488 ± 3.207	488.373 ± 42.322	360.929
*N*_RoB_	14.012 ± 3.258	12.232 ± 0.188	9.070 ± 1.069	69.358 ± 0.762	44.671
*N*_OB_	9.694 ± 1.745	12.828 ± 0.380	n.a.	“20.909”	n.a.
*O*_BR_	78.594 ± 7.733	103.349 ± 15.610	46.361 ± 0.527	620.622 ± 37.616	413.574
*O*_CX_	18.131 ± 0.071	32.828 ± 5.773	19.712 ± 4.369	316.979 ± 28.785	166.660
*O*_HP_	8.097 ± 1.176	7.659 ± 0.555	1.658 ± 0.246	49.654 ± 15.244	16.877
*O*_CxT_	26.229 ± 1.104	40.486 ± 5.217	21.370 ± 4.614	366.620 ± 13.520	183.540
*O*_CB_	23.368 ± 1.279	34.658 ± 15.801	8.155 ± 0.813	91.005 ± 30.180	77.571
*O*_RoB_	28.997 ± 7.906	28.204 ± 5.408	16.835 ± 3.276	162.985 ± 6.105	152.467
*O*_OB_	4.919 ± 1.108	14.776 ± 5.204	n.a.	14.791	n.a.
*DN*_BR_	124,661 ± 5575	64,104 ± 5587	80,519 ± 6978	45,576 ± 8325	39,413
*DN*_CX_	46,272 ± 426	28,213 ± 4497	51,070 ± 2260	18,726 ± 4212	13,272
*DN*_HP_	77,004 ± 17,012	23,164 ± 975	33,628 ± 6273	21,712 ± 2982	11,450
*DN*_CxT_	54,644 ± 6098	27,236 ± 3416	48,932 ± 1004	19,134 ± 3955	13,098
*DN*_CB_	531,494 ± 10,651	362,537 ± 15,780	409,687 ± 18,667	242,415 ± 46,950	188,180
*DN*_RoB_	34,520 ± 2702	13,696 ± 547	31,616 ± 5124	16,436 ± 2556	13,423
*DN*_OB_	194,678 ± 4,037	80,805 ± 2181	n.a.	73,110	n.a.
*DO*_BR_	75,454 ± 1486	42,206 ± 4518	57,227 ± 2452	37,317 ± 5542	32,305
*DO*_CX_	51,102 ± 1096	33,653 ± 3322	50,301 ± 8149	34,590 ± 3820	24,401
*DO*_HP_	71,995 ± 8477	28,244 ± 276	33,005 ± 2294	39,186 ± 2271	23,247
*DO*_CxT_	55,693 ± 138	32,550 ± 2661	48,146 ± 6674	35,203 ± 3461	24,291
*DO*_CB_	139,028 ± 5956	110,153 ± 41,831	96,849 ± 5069	46,365 ± 19,711	40,444
*DO*_RoB_	70,967 ± 8566	31,716 ± 6830	58,880 ± 13,983	38,353 ± 4138	45,813
*DO*_OB_	97,872 ± 2587	91,369 ± 27,555	n.a.	51,715	n.a.
*O/N*_BR_	0.607 ± 0.039	0.657 ± 0.013	0.713 ± 0.031	0.824 ± 0.029	0.820
*O/N*_CX_	1.104 ± 0.013	1.205 ± 0.074	0.980 ± 0.116	1.897 ± 0.223	1.838
*O/N*_HP_	1.008 ± 0.332	1.221 ± 0.039	1.004 ± 0.119	1.825 ± 0.146	2.030
*O/N*_CxT_	1.032 ± 0.119	1.202 ± 0.053	0.982 ± 0.116	1.883 ± 0.208	1.855
*O/N*_CB_	0.261 ± 0.005	0.299 ± 0.102	0.236 ± 0.002	0.182 ± 0.046	0.215
*O/N*_RoB_	2.048 ± 0.088	2.300 ± 0.407	1.839 ± 0.144	2.351 ± 0.114	3.413
*O/N*_OB_	0.503 ± 0.024	1.141 ± 0.372	n.a.	0.707	n.a.

*Cellular composition of the five Afrotherian species. All masses in grams. Numbers of cells in millions. M_BD_, body mass; M_BR_, brain mass; M_CX_, mass of the cerebral cortex (excluding the hippocampus); M_HP_, mass of the hippocampus; M_CXT_, mass of the cerebral cortex including the hippocampus; M_CB_, mass of the cerebellum; M_ROB_, mass of the rest of the brain; M_OB_, mass of the olfactory bulb; N, number of neurons; O, number of other (non-neuronal) cells; DN, density of neurons (neurons/mg); DO, density of other cells (other cells/mg); O/N, ratio between numbers of other cells and neurons*.

### Relative distribution of mass and neurons

The relative size of each brain structure (expressed as a percentage of total brain mass) does not vary significantly with total brain mass across the five afrotherian species studied (*p* > 0.18 for all correlations). On average, we find that the cerebral cortex (without the hippocampus) corresponds to 45.2 ± 2.9% of brain mass, or to 53.8 ± 2.2% with the hippocampus; the cerebellum amounts to 13.1 ± 1.1% of brain mass; the hippocampus, to 8.4 ± 1.2% of brain mass; and the rest of the brain, to 32.5 ± 2.8% of brain mass.

The relative number of brain neurons found in each structure also does not vary significantly with brain mass across the five afrotherian species (all correlations, *p* > 0.28). As in other mammalian species, the cerebellum of these afrotherians is the structure that houses most of the brain neurons, with on average 66.2 ± 3.6% of all brain neurons despite its much smaller relative mass. This is followed by the cerebral cortex (without the hippocampus) with 24.6 ± 2.0% of all brain neurons, the rest of the brain, with 10.2 ± 1.1%, and the hippocampus, with 3.3 ± 0.6% of all brain neurons. The golden mole appears to be an outlier, with the smallest relative number of neurons in the cerebellum (only 52.77% of its total brain neurons, while the other species average 70%). The relative mass of each structure shows no correlation to the relative number of neurons in each structure (*p* > 0.18), with the exception of the hippocampus (ρ = 1.0, *p* < 0.0001).

### Neuronal and non-neuronal scaling rules

**Figure 3:**
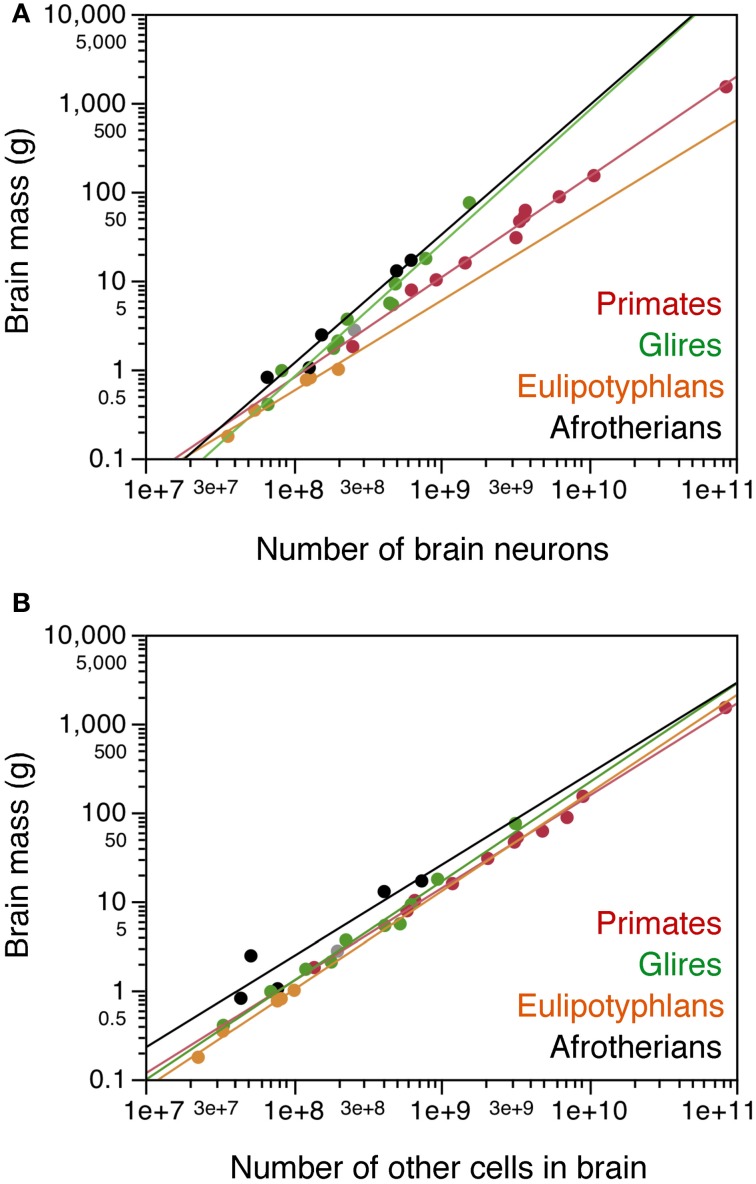
**Cellular scaling rules for afrotherian brains**. Average brain mass for each species is plotted as a function of its total number of neurons **(A)** or other, non-neuronal cells in the brain **(B)**. Brain does not include olfactory bulb. Afrotherian species shown in black, primates in red, scandentia in gray, glires in green, and eulipotyphlans in orange. Data from Herculano-Houzel et al. (2006, 2007, 2011); Azevedo et al. (2009); Sarko et al. (2009); Gabi et al. (2010). Power functions for each group are plotted. Exponents for brain mass × numbers of neurons are 1.451 ± 0.163 (afrotherians), 1.499 ± 0.107 (Glires), 1.016 ± 0.108 (eulipotyphlans), 1.130 ± 0.036 (primates with Scandentia). Exponents for brain mass × numbers of other cells are 1.025 ± 0.218 (afrotherians), 1.114 ± 0.049 (Glires), 1.105 ± 0.082 (eulipotyphlans), 1.040 ± 0.020 (primates with Scandentia).

Across the afrotherian species studied, total brain mass can be described as a power function of its number of neurons with an exponent of 1.451 ± 0.163 (*p* = 0.0030), indicating that the brain as a whole gains mass more rapidly than it gains neurons (Figure 3A). The distribution of brain mass and total number of neurons in afrotherians overlaps with that for glires (rodents and lagomorphs), which has a similar exponent of 1.499 ± 0.107 (Herculano-Houzel et al., 2011). As a result, afrotherians and rodents with similar brain masses also have similar numbers of neurons (Figure 3A). In contrast, afrotherians have much larger brains than eulipotyphlans with similar numbers of neurons (Figure 3A, compare black and orange points), and also gain brain mass faster than insectivores as they gain neurons (insectivores, exponent 1.016 ± 0.108).

Brain mass in the afrotherians studied varies as a power function of the number of other cells in the brain (Figure 3B) with an exponent of 1.025 ± 0.218 (*r*^2^ = 0.880, *p* = 0.0183), but can be described even better as a linear function of the number of other cells in the brain (*r*^2^ = 0.953; *p* = 0.0044). The distribution of brain mass and number of other cells overlaps among afrotherians, Glires, eulipotyphlans and primates (Figure 3B).

**Figure 4:**
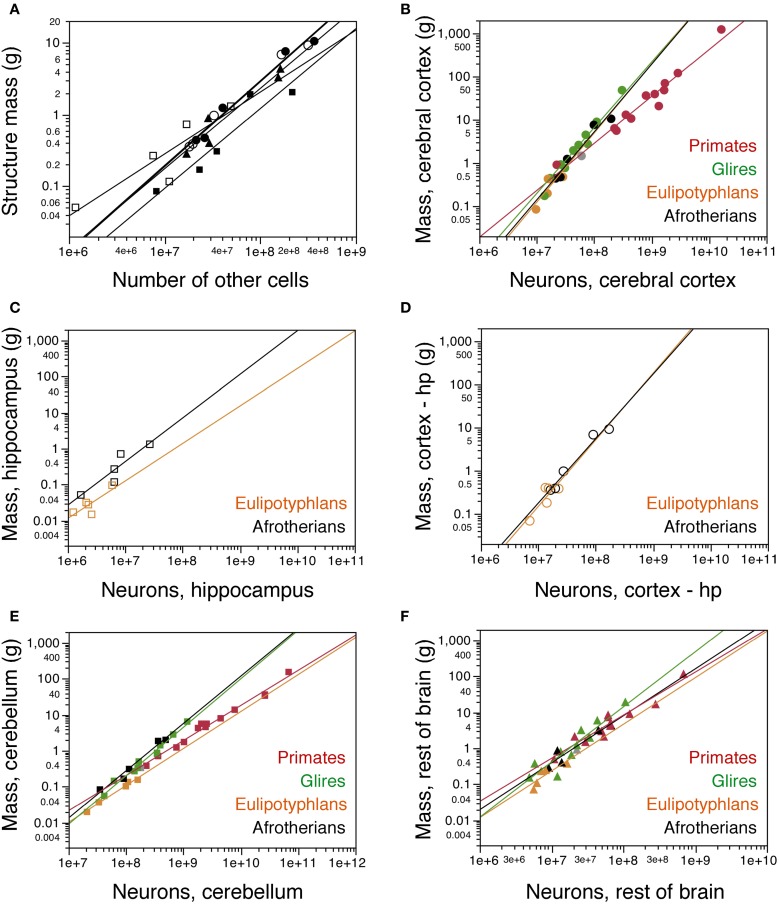
**Cellular scaling rules for afrotherian brain structures. (A)** Average mass of each brain structure is plotted for each afrotherian species as a function of its total number of other (non-neuronal) cells. Power functions are plotted separately for each structure, and have similar exponents of 1.179 ± 0.091 (cerebral cortex without hippocampus, open circles), 1.182 ± 0.109 (cerebral cortex with hippocampus, filled circles), 1.112 ± 0.214 (cerebellum, filled squares), 0.862 ± 0.239 (hippocampus, open squares), and 1.119 ± 0.168 (rest of brain, triangles). **(B–F)** Average mass of each brain structure plotted for each species as a function of its total number of neurons. **(B)** cerebral cortex including hippocampus; **(C)** hippocampus; **(D)** cerebral cortex without hippocampus; **(E)** cerebellum; **(F)** rest of brain. Afrotherian species shown in black, primates in red, scandentia in gray, glires in green, and eulipotyphlans in orange. Data from Herculano-Houzel et al. (2006, 2007); Herculano-Houzel et al. (2011); Azevedo et al. (2009); Sarko et al. (2009); Gabi et al. (2010). Power functions for each group are plotted. Exponents for structure mass × numbers of neurons are: Cerebral cortex with hippocampus **(B)**, 1.540 ± 0.199 (afrotherians), 1.699 ± 0.096 (Glires), 1.598 ± 0.618 (eulipotyphlans), 1.087 ± 0.074 (primates). Hippocampus **(C)**, 1.121 ± 0.312 (afrotherians), 1.041 ± 0.422 (eulipotyphlans). Cerebral cortex without hippocampus **(D)**, 1.497 ± 0.157 (afrotherians), 1.546 ± 0.575 (eulipotyphlans). Cerebellum **(E)**, 1.316 ± 0.130 (afrotherians), 1.305 ± 0.072 (Glires), 0.976 ± 0.036 (eulipotyphlans), 1.028 ± 0.084 (primates). Rest of brain **(F)**, 1.305 ± 0.246 (afrotherians), 1.297 ± 0.476 (eulipotyphlans), 1.198 ± 0.112 (primates), 1.533 ± 0.203 (Glires).

Similarly as found for the whole brain, the mass of each brain division in afrotherians can be described as a power function of its number of other cells with an exponent close to unity, indicative of near-linearity (Cx: 1.179 ± 0.091; Cb: 1.112 ± 0.214; RoB: 1.119 ± 0.168; Hp: 0.862 ± 0.239; CxT: 1.182 ± 0.109; Figure 4A).

The mass of the cerebral cortex and of the cerebellum vary as power functions of the number of neurons in the structure with exponents significantly above linearity (Cx: 1.497 ± 0.157, *p* = 0.0024; CxT: 1.540 ± 0.199, *p* = 0.0045; Cb: 1.316 ± 0.130, *p* = 0.0020; Figures 4B,D,E). The large exponents indicate that, across afrotherian species, the cerebral cortex and the cerebellum gain mass faster than they gain neurons. The exponent of 1.540 for the cerebral cortex (including the hippocampus, as in previous studies) overlaps with the exponents that apply to glires (1.699 ± 0.096) and Eulipotyphla (1.598 ± 0.618) but not with the nearly linear exponent of 1.087 ± 0.074 that describes the relationship for Primata (Figure 4B). In fact, there is considerable overlap for the cerebral cortex (including the hippocampus) datapoints across Glires, Eulipotyphla, and Afrotheria (Figure 4B), and for the cerebral cortex minus hippocampus across Eulipotyphla and Afrotheria (Figure 4D). The overlap indicates that the scaling rules that relate cortical mass to number of neurons are similar across the three orders, but different from primates.

Analyzing the same variables for the cerebellum, we again find that the exponent for Afrotheria (1.316 ± 0.130) overlaps with that for Glires (1.305 ± 0.072) but not with those for Primata and Eulipotyphla, which are both close to one (1.028 ± 0.084 and 0.976 ± 0.036, respectively; Figure 4E). The distribution of cerebellar mass and number of neurons observed in afrotherians also overlaps with Glires, indicating that these two orders also share a neuronal scaling rule for the cerebellum. In comparison, for a similar number of cerebellar neurons, Afrotheria and Glires have a much larger cerebellar mass than Eulipotyphla (Figure 4E).

The exponents for rest of brain and hippocampus cannot be statistically distinguished from unity (RoB: 1.305 ± 0.246; Hp: 1.121 ± 0.312; Figures 4C,F). The mass of the hippocampus scales with its number of neurons raised to similar exponents in Afrotheria and Eulipotyphla [1.041 ± 0.422; (Sarko et al., 2009)]. However, the intercepts of the functions are different, such that for a similar number of neurons, the afrotherian hippocampus has a larger mass than the eulupotyphlan hippocampus (Figure 4C).

The rest of brain appears to gain neurons in a similar way across afrotherians, Glires, eulipotyphla, and primates, with largely overlapping scattered distributions and exponents that are statistically indistinguishable (Figure 4F).

The olfactory bulb was only available for three afrotherian species (the two elephant shrews and the rock hyrax; Table 1). Although three species are not enough to determine the neuronal scaling rules for the afrotherian olfactory bulb, the distribution of mass and number of neurons in the olfactory bulb was found to be similar across these afrotherians species and Glires, whereas both orders have larger olfactory bulbs than Eulipotyphla for a similar number of neurons.

### Cell densities

**Figure 5:**
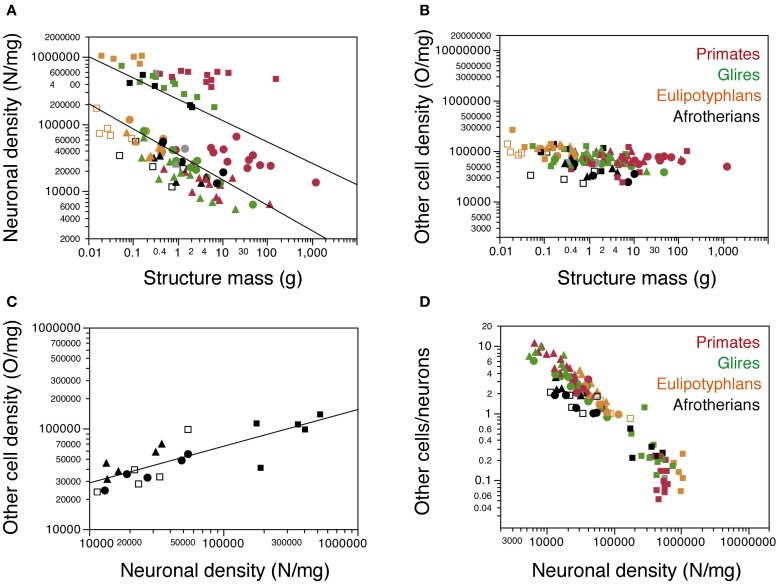
**Cellular density variation. (A, B)** average neuronal density [in number of neurons per mg of tissue; **(A)**] and average density of other cells [in number of other cells per mg of tissue; **(B)**] are plotted for each structure in each species, with a similar Y-axis for comparison. Afrotherian species are plotted in black, glires in green, primates in red, scandentia in gray, and insectivores in orange. For clarity, only the significant power functions for afrotherians are plotted in **(A)** (cerebral cortex with hippocampus, exponent −0.378 ± 0.082; cerebellum, exponent −0.317 ± 0.070). No brain structure in no mammalian order examined here exhibits a significant correlation between other cell density and structure mass. **(C)** variation in other cell density plotted as a function of neuronal density for afrotherian brain structures only. The function plotted applies to the ensemble of all structures (exponent, 0.363 ± 0.060). **(D)** variation of the O/N ratio, that is, between numbers of other cells and numbers of neurons in each brain structure of each species, plotted as a function of neuronal density in the same structure. Filled circles, cerebral cortex (with hippocampus); open squares, hippocampus; filled squares, cerebellum; filled triangles, rest of brain. Data from Herculano-Houzel et al. (2006, 2007, 2011); Azevedo et al. (2009); Sarko et al. (2009); Gabi et al. (2010).

As observed for other groups of mammals, neuronal density varies considerably more than other cell density across structures (Figures 5A,B). Neuronal density in the cerebral cortex ranges from 13,272 neurons/mg in the tree hyrax to 51,070 neurons/mg in the golden mole; in the hippocampus, from 11,450 neurons/mg in the tree hyrax to 54,601 neurons/mg in the elephant shrew; in the cerebellum, from 175,610 neurons/mg in the rock hyrax to 531,494 neurons/mg in the golden mole; and in the rest of brain, from 13,423 neurons/mg in the tree hyrax to 34,520 neurons/mg in the eastern rock elephant shrew (Table 1, DN; Figure 5A). The density of other cells, in comparison, ranges from 23,247 cells/mg in the hippocampus of the tree hyrax to 139,028 cells/mg in the cerebellum of the elephant shrew (Table 1, DO; Figure 5B).

Consistently with the faster increase in structure mass than in number of neurons in the afrotherian cerebral cortex and cerebellum, neuronal density decreases significantly in these structures as a power function of increasing structure mass (exponents, Cx: −0.351 ± 0.069, *p* = 0.0146; CxT, −0.378 ± 0.082, *p* = 0.0191; Cb: −0.317 ± 0.070, *p* = 0.0201; Figure 5A). In agreement with a linear scaling of hippocampal and RoB mass with number of neurons, neuronal density does not vary significantly with structure mass in either region (Hp, *p* = 0.1815; RoB, *p* = 0.1049). No brain structure exhibits a significant correlation between other cell density and structure mass (Cx, *p* = 0.0851; CxT, *p* = 0.1118; Hp, *p* = 0.5739; Cb, *p* = 0.3709; RoB, *p* = 0.2869). Despite the lack of a significant correlation between other cell density and structure mass (Figure 5B), variations in other cell density are a significant power function of variations in neuronal density across all structures (with an exponent of 0.363 ± 0.060, *p* < 0.0001; Figure 5C), as well as within the cerebral cortex and rest of brain (exponents of 0.514 ± 0.091, *p* = 0.0112, and 0.621 ± 0.188, *p* = 0.0455, respectively).

The ratio between the numbers of other cells and neurons in each structure (the O/N ratio), which approximates the glia/neuron ratio, varies between 0.215 and 3.413 across structures and species in these afrotherians (Figure 5D, black datapoints). The O/N ratio varies as a common power function of neuronal density across all structures with an exponent of −0.671 ± 0.058 (*p* < 0.0001), and also separately within the cerebral cortex (without hippocampus: exponent −0.484 ± 0.093, *p* = 0.0140; with hippocampus: exponent −0.486 ± 0.086, *p* = 0.0148). As for the covariation of other cell density and neuronal density, the correlation between variations in O/N ratio and in neuronal density is stronger for the entire dataset (that is, across brain structures) than within individual structures. Interestingly, despite the similar decrease in O/N ratio with increasing neuronal densities across structures and species, the O/N ratio in the cerebral cortex, hippocampus and rest of brain is about 2 times smaller in afrotherians than in glires, primates, and eulipotyphlans for similar neuronal densities (Figure 5D).

### Correlations across structures

**Figure 6:**
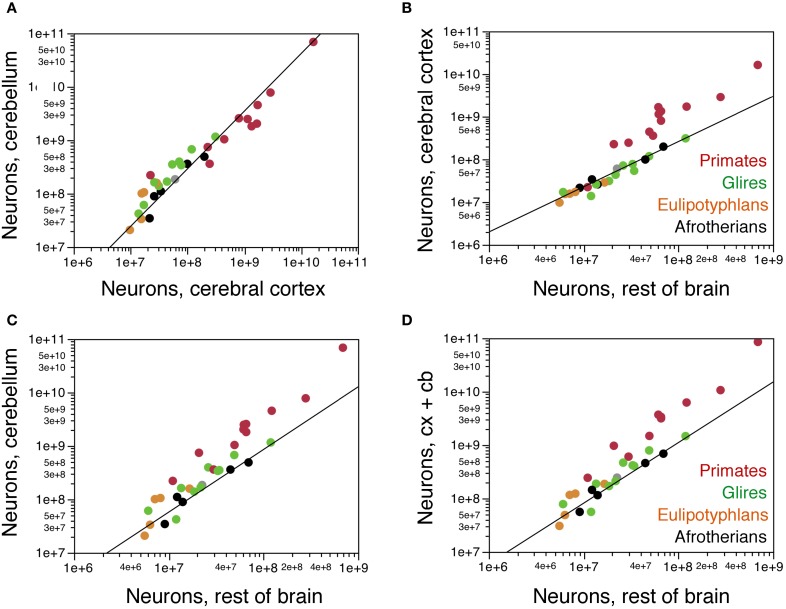
**Covariation in numbers of neurons in brain structures across species. (A)** Numbers of neurons in the cerebellum vary as a common power function of numbers of neurons in the cerebral cortex of exponent 1.079 ± 0.200 (plotted), or as a linear function of slope 4.164 ± 0.088, that is shared by afrotherians, Glires, eulipotyphlans, and primates. **(B)** Numbers of neurons in the cerebral cortex vary as a common function of numbers of neurons in the rest of brain that is shared by afrotherians, Glires, and eulipotyphlans, but not primates, with a linear coefficient of 2.668 ± 0.091. **(C)** Numbers of neurons in the cerebellum also vary as a common function of numbers of neurons in the rest of brain that is shared by afrotherians, Glires, and eulipotyphlans, but not primates, with a linear coefficient of 9.646 ± 0.671. **(D)** Combined numbers of neurons in the cerebral cortex and cerebellum vary as a common function of numbers of neurons in the rest of brain that is shared by afrotherians, Glires, and eulipotyphlans, but not primates, with a linear coefficient of 12.314 ± 0.666.

Across these afrotherian species, the cerebral cortex and cerebellum gain neurons concertedly (Figure 6A), as found previously across primates, rodents and eulipotyphlans (Herculano-Houzel, 2010). The relationship between numbers of neurons in the two structures is better described as a linear (*r*^2^ = 0.933, *p* = 0.0076) than as a power function (exponent 1.079 ± 0.200, *r*^2^ = 0.906, *p* = 0.0125) and overlaps with that for other mammalian orders (Figure 6A). In fact, the addition of afrotherians to the previous dataset does not alter significantly alter the linear coefficient that relates numbers of neurons in the cerebellum to numbers of neurons in the cerebral cortex (without Afrotheria: 4.174 ± 0.096, *r*^2^ = 0.987, *p* < 0.0001; with Afrotheria: 4.164 ± 0.088, *r*^2^ = 0.987, p<0.0001).

The cerebral cortex gains neurons in relation to the rest of brain at the same rate in Afrotheria as in Glires and Eulipotyphla (Figure 6B). The relationship between numbers of neurons in the two structures is again best described as linear in Afrotheria (*r*^2^ = 0.975, *p* = 0.0017; power function, exponent 1.059 ± 0.110, *r*^2^ = 0.968, *p* = 0.0024) as well as in Glires and Eulipotyphla combined (*r*^2^ = 0.984, *p* < 0.0001; power function, exponent 1.043 ± 0.075, *r*^2^ = 0.937, *p* < 0.0001). The distribution of datapoints overlaps among Afrotheria, Glires, and Eulipotyphla, and is best described for the three clades jointly by a linear function with a coefficient of 2.668 ± 0.091 (*r*^2^ = 0.980, *p* < 0.0001), similar to the coefficient that describes Glires and Eulipotyphla alone (2.622 ± 0.094, *r*^2^ = 0.984, *p* < 0.0001; Figure 6B). This is significantly different from the linear coefficient of 22.998 ± 1.676 (*r*^2^ = 0.950, *p* < 0.0001) that applies to primates (Figure 6B). Thus, afrotherians, like Glires and Eulipotyphla, have a nearly 10-fold smaller ratio of neurons in the cerebral cortex to neurons in the rest of brain than do primates, but that ratio is similar in Afrotheria, Glires, and Eulipotyphla.

The cerebellum also gains neurons in relation to the rest of the brain at the same rate in Afrotheria as in Glires and Eulipotyphla (Figure 6C), as expected from the linear relationship between numbers of neurons in the cerebellum and cerebral cortex across all species. The relationship between numbers of neurons in the two structures is also best described as linear in Afrotheria (*r*^2^ = 0.981, *p* = 0.0011; power function, exponent 1.172 ± 0.194, *r*^2^ = 0.924, *p* = 0.0091) as well as in Glires and Eulipotyphla combined (*r*^2^ = 0.950, *p* < 0.0001; power function, exponent 1.180 ± 0.135, *r*^2^ = 0.854, *p* < 0.0001). The distribution of data points again overlaps among Afrotheria, Glires, and Eulipotyphla, and is best described across the three orders by a linear function with a coefficient of 9.646 ± 0.671 (*r*^2^ = 0.920, *p* < 0.0001), similar to the coefficient that describes Glires and Eulipotyphla alone (10.308 ± 0.658, *r*^2^ = 0.950, *p* < 0.0001; Figure 6C). This is significantly different from the linear coefficient of 97.862 ± 9.126 (*r*^2^ = 0.927, *p* < 0.0001) that applies to primates (Figure 6C). Thus, afrotherians, like Glires and Eulipotyphla, have a 10-fold smaller ratio of neurons in the cerebellum to neurons in the rest of brain than primates, but that ratio is similar in Afrotheria, Glires and Eulipotyphla.

The combined number of neurons in the cerebral cortex and cerebellum again scales as a linear function of the number of neurons in the rest of brain that overlaps among Afrotheria, Glires, and Eulipotyphla (joint coefficient, 12.314 ± 0.666, *r*^2^ = 0.950, *p* < 0.0001; Figure 6D) and differs from the coefficient that applies to primates by a factor of 10 [120.811 ± 10.825, *r*^2^ = 0.933, *p* < 0.0001; (Herculano-Houzel, 2011b)]. Thus, Afrotheria share with Glires and Eulipotyphla, but not with primates, the rate at which their cerebral cortex and cerebellum gain neurons in respect to the rest of brain.

### Gyrification

**Figure 7:**
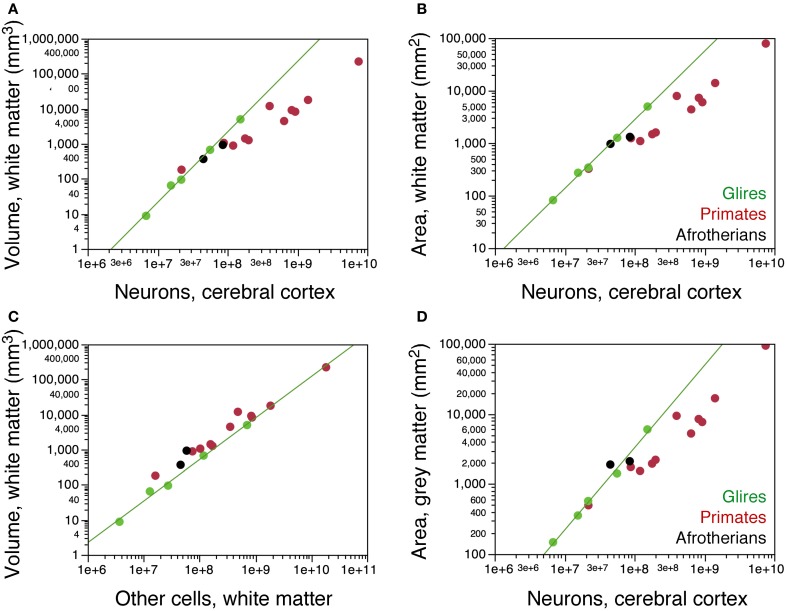
**Scaling of the subcortical white matter. (A)** Variation in the volume of the subcortical white matter as a function of numbers of neurons in the cerebral cortex in the two hyrax species (afrotherians, black), rodents (green) and primates (red). The hyraxes overlap with the power function that applies to rodents (exponent, 2.009 ± 0.065). **(B)** Variation in the surface area of the subcortical white matter (that is, the surface of the gray-white matter interface) as a function of numbers of neurons in the cerebral cortex. The hyraxes overlap with the power function that applies to rodents (exponent, 1.308 ± 0.038). **(C)** Variation in the volume of the subcortical white matter as a function of numbers of other cells that it contains. The hyraxes have a larger white matter volume than expected for its number of other cells compared to rodents (exponent, 1.185 ± 0.060). **(D)** Variation in the volume of the cortical gray matter as a function of its numbers of neurons. The hyraxes overlap with the power function that applies to rodents (exponent, 1.174 ± 0.051), but notice that the rock hyrax (black circle on the right) has twice as many neurons in the cortex as the tree hyrax (black circle, left). Data from Ventura-Antunes et al. (2013). Data on the cortical surface areas and volumes were not available for eulipotyphlans and the smaller afrotherians.

We next examined how the distribution of neurons per cortical surface and in relationship to the white matter compares across the two gyrencephalic afrotherians in our sample and other gyrencephalic mammals. No eulipotyphlans were included in this analysis, as all eulipotyphlans in our sample are lissencephalic, and data on their cortical surface areas and volumes were not available. Both hyrax species overlap with glires in the relationship between the volume of the white matter and the number of neurons in the gray matter (Figure 7A). In contrast, both hyrax species diverge from glires in that the hyraxes have larger white matter volumes for a similar number of other cells in the white matter (Figure 7C), which are assumed to be proportional to the total length of myelinated axons in the white matter. This suggests a larger average caliber of the myelinated fibers in the white matter in afrotherians than in rodents (see model in Mota and Herculano-Houzel, 2012).

Both hyrax species also appear to overlap with rodents in the relationship between the surface area of the white matter (Figure 7B) or of the gray matter (Figure 7D) and the number of neurons in the gray matter. However, the rock hyrax has twice as many neurons than the tree hyrax in a cerebral cortex of similar gray and white matter surface areas (Figures 7B,D). As a consequence, the average number of neurons beneath 1 mm^2^ of cortical surface is much smaller in the tree hyrax (at 23,808 N/mm^2^) than in the rock hyrax (40,521 N/mm^2^), and also than in a rodent with a similar number of cortical neurons to the tree hyrax (the agouti, with 39,868 N/mm^2^). These findings, although limited to two species, raise the possibility that the distribution of neurons under the cortical surface in afrotherians does not follow the same rules as in Rodents.

**Figure 8:**
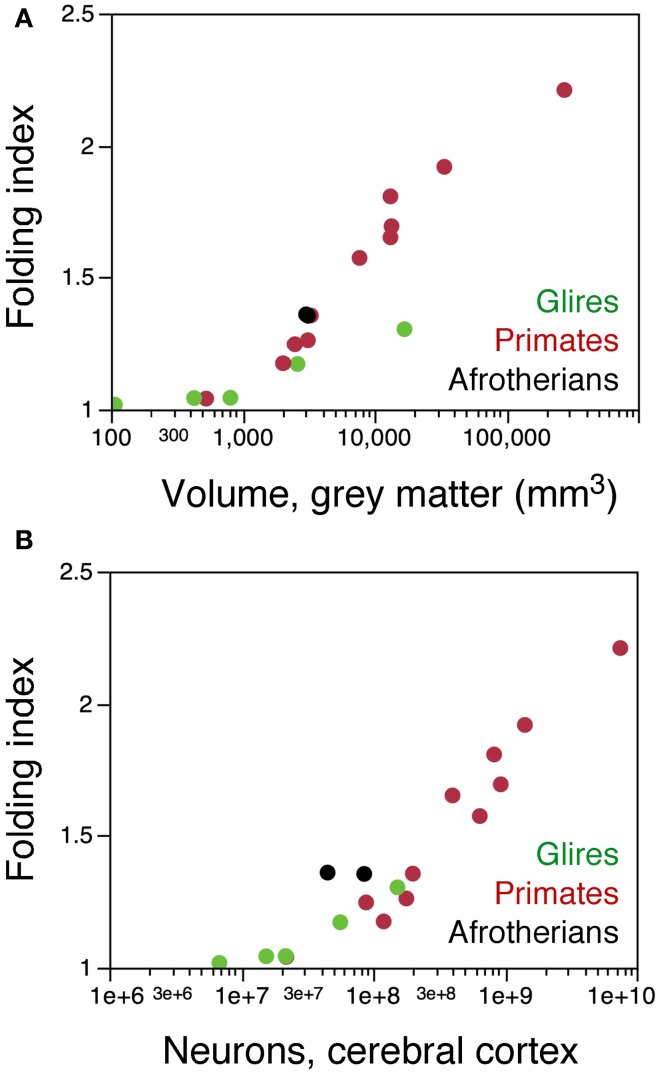
**The afrotherian cortex is folded differently from that of rodents and primates. (A)** folding index of the cerebral cortex (ratio between the total surface area and the exposed surface area of the cortex) plotted as a function of the volume of the cortical gray matter for rodents (green), primates (red), and the two hyraxes (black). Notice that the two hyraxes appear to overlap with primates, but not rodents. **(B)** folding index of the cerebral cortex plotted as a function of the number of cortical neurons in each species. For similar numbers of cortical neurons, the cortex of the two hyraxes is more folded that the cortex of both primates and rodents. Data from Ventura-Antunes et al. (2013). Eulipotyphla and the smaller Afrotheria are lissencephalic, and data on their cortical surface areas and volumes were not available.

Further evidence of a different arrangement of neurons in the afrotherian cortical surface compared to rodents is the degree of cortical gyrification. When the folding index is plotted as a function of cortical gray matter surface, Afrotheria overlap with primates, not with rodents (Figure 8A). However, plotting the folding index as a function of cortical neurons reveals that the two gyrencephalic afrotherians in our sample, the tree hyrax and the rock hyrax, have a much larger folding index than both rodents and primates with a similar number of cortical neurons (Figure 8B). These findings support our previous observation that gyrencephaly is not a simple function of the number of neurons or of a larger cortical surface across mammalian species (Ventura-Antunes et al., 2013), and suggest that neurons are indeed distributed across the cortical surface differently across afrotherians, Rodents and Primates.

Finally, the neuronal scaling rules that apply to the current sample of afrotherians can be used to predict the neuronal composition of other afrotherian brains, such as the African elephant. With a cerebellar volume of 930 ml (Maseko et al., 2012) and a total (gray+white matter) cortical volume of 2491 cm^3^ (Hofman, 1985), we predict that the African elephant has 151 billion neurons in the cerebellum (that is, more than twice the number of neurons in the human cerebellum), but only 5.4 billion neurons in its cerebral cortex, that is, less than a third of the neurons in the human cerebral cortex (Azevedo et al., 2009).

## Discussion

Here we find that the brains of small afrotherian mammals gain neurons more slowly than they gain mass, with decreasing neuronal densities in the cerebral cortex and cerebellum that indicate an increase in the average size (cell body plus all arbors) of the neurons in these structures. In contrast, the hippocampus and rest of brain structures do not show a significant change in neuronal density with increasing structure mass.

Across the afrotherian species studied, the cerebral cortex and cerebellum gain neurons in concert, sharing the same relationship that was found previously across primates, rodents and eulipotyphlans (Herculano-Houzel, 2010). In contrast, the cerebral cortex and the cerebellum gain neurons in relation to the rest of brain at the same rate in Afrotheria as in Glires and Eulipotyphla, which is more slowly than the rate found in primates (Herculano-Houzel, 2011b).

Overall, we find that the neuronal scaling rules that apply to building the entire cortical mass as well as the cerebellum of Afrotheria overlap with those that apply to Glires, but not Eulipotyphla. The cerebral cortex of afrotherians does overlap with both Glires and Eulipotyphla in the relationship between cortical mass and number of neurons as well as in neuronal densities for a given cortical mass. However, the afrotherian cerebellum overlaps in the relationship between structure mass and number of neurons only with Glires, but not Eulipotyphla, and the afrotherian hippocampus and olfactory bulb are larger than those of Eulipotyphla with a similar number of neurons. These findings raise the possibility that the neuronal scaling relationships that apply to the brain mass of Glires already applied to the ancestor shared with afrotherians, a possibility that is further supported by our unpublished finding of similar neuronal scaling relationships shared by Artiodactyla as well (Siqueira, Manger and Herculano-Houzel, pers. comm.).

In summary, we find that relationships for numbers of neurons across structures are shared among Afrotheria, Eulipotyphla, and Glires, but not primates, while relationships between structure mass and number of neurons are shared among Afrotheria and Glires, but not Eulipotyphla or Primates. In contrast, the relationship between number of cortical neurons and cortical gyrification is shared neither between Afrotheria and Glires, nor between Afrotheria and Primates (Ventura-Antunes et al., 2013). This shows for the first time that while volume relationships for the cerebral cortex may be shared across mammalian phylogenetic groupings, as in the case of Afrotheria and Glires, the distribution of those neurons under the surface may still differ dramatically. We suggest that the different distribution of neurons per cortical surface area in the face of a similar distribution of neurons per cortical volume is related to factors such as the fraction of cortical neurons that are connected through the white matter and the average caliber of the fibers in the white matter, which we have shown to impact the extent of the cortical surface in a model of cortical folding (Mota and Herculano-Houzel, 2012).

### Non-neuronal scaling

Here we find that the non-neuronal scaling rules that apply to Glires, Primates and Eulipotyphla are also shared by the Afrotheria. This shared relationship suggests that the rules that govern the addition of non-neuronal cells to different structures of mammalian brains date back to at least 100 million years, prior to the divergence of Afrotheria, at the base of the eutherian clade. The evolutionarily conserved nature of the non-neuronal scaling rules, which occurs in parallel with divergent neuronal scaling rules, suggests that non-neuronal cells, which are presumed to be glial in their vast majority, are crucial to tissue physiology and must comprise a specific proportion of brain tissue in a very precise manner that does not allow for significant variation across brain structures or across species. This in turn leads to the question of how far back the non-neuronal scaling rules go in the history of brain evolution. The next logical step will be to investigate the brains of non-eutherian mammals - marsupials and monotremes, which diverged from other mammals even earlier than Afrotheria—in order to see if their brains also conform to the so-far universal scaling rules found. Our preliminary data suggest that the non-neuronal scaling rules that we suggest to be universal among mammals also apply to birds (Seweryn Olckzewicz, Herculano-Houzel and Pavel Nemec, pers. comm.). While neuronal scaling rules are much more variable across structures and mammalian orders than non-neuronal scaling rules, our finding that some neuronal scaling rules are shared between afrotherians and rodents, and that some features are additionally shared with insectivores and primates, raise the interesting possibility that these shared characteristics applied to the common eutherian ancestor. The clade-specific characteristics that relate to the distribution of neurons along the surface of the cerebral cortex and to its degree of gyrification, in turn, suggest that these characteristics compose a suite of features that may have defined different mammalian groups in evolution.

### Afrotheria are not insectivora

On the basis of their shared morphology and ecology, small afrotherians were long considered to group as Insectivora with other insectivorous animals today placed in the order Eulipotyphla (Stanhope et al., 1998). Our data show that, despite the shared ecological traits, small afrotherians show larger brain structures (cerebellum, hippocampus and olfactory bulb) than Eulipotyphla for similar numbers of neurons, and actually only share with Eulipotyphla those features that both orders also share with Glires, such as the neuronal scaling rules for the cerebral cortex and the rate of addition of neurons to the cerebral cortex and cerebellum in relation to the rest of the brain. Even elephant shrews, which have recently been suggested to be encephalized compared to eulipotyphlans (Kaufman et al., 2013) do not seem to reach the number of neurons of Eulipotyphla of the same size (Figure 5). The different neuronal scaling rules for Afrotheria and Eulipotyphla indicate that, in this case, divergent phylogenetic histories with their accompanying phylogenetic constraints have been strong enough to override the possible effects of shared ecological pressures in the cellular composition of these brains. However, it is still possible that selective pressures shape the brain in ways other than the ones investigated here, such as at the level of connectivity or functional properties of individual cortical areas.

One limitation of the present study is that it addresses only the lower part of the range of sizes present in extant Afrotheria. This could be resolved with the addition of aardvarks, sirenians and elephants, which are interesting not only because of their complementary brain size—which would put to a test the rules determined here for Afrotheria -, but also because of specific adaptations that must be reflected in the structure of their brains. Afrotheria is a diverse superorder with relatively few but varied species, which might also explain the differences found between the tree hyrax and the rock hyrax. Despite the similar common names, the rock hyrax and the tree hyrax belong to different genus (*Procavia* and *Dendrohyrax*) that appear to be at opposite ends within the hyracoid radiation (Skinner and Chimimba, 2005). They also have different habits: while the rock hyrax is diurnal, social (living in groups of up to 80 individuals) with seasonal mating, and an opportunistic feeder, the tree hyrax is nocturnal, predominantly solitary with no particular breeding season, and a selective browser (Skinner and Chimimba, 2005). In the context of this diversity, it is still remarkable that the five species analyzed here conform to the neuronal scaling rules that apply to glies. As a result, by extrapolating from the neuronal scaling rules found here, we were able to predict the neuronal composition of the brain of the African elephant at 151 billion cerebellar neurons, which is more that twice the number of neurons in the human cerebellum. This agrees with both the large absolute and relative size of the elephant cerebellum (Maseko et al., 2012). In contrast, we predict a total of only 5.4 billion neurons in the cerebral cortex of the African elephant, which is less than a third of the neurons found in the human cerebral cortex (Azevedo et al., 2009), with a predicted low average neuronal density close to what has been found in select locations in the Asian elephant cerebral cortex (Tower, 1954). We are in the process of determining whether the scaling rules encountered for small Afrotherians also apply to the elephant brain, and of testing the hypothesis that the human brain has more neurons in the cerebral cortex than the elephant brain, which we have proposed to be the basis for the remarkable cognitive abilities of the human brain (Herculano-Houzel, 2009).

### Conflict of interest statement

The authors declare that the research was conducted in the absence of any commercial or financial relationships that could be construed as a potential conflict of interest.
